# Correction: Li, et al. Conserved miR396b-GRF Regulation Is Involved in Abiotic Stress Responses in Pitaya (*Hylocereus polyrhizus*). *Int. J. Mol. Sci.* 2019, *20*, 2501

**DOI:** 10.3390/ijms20194795

**Published:** 2019-09-27

**Authors:** A-Li Li, Zhuang Wen, Kun Yang, Xiao-Peng Wen

**Affiliations:** The Key Laboratory of Plant Resources Conservation and Germplasm Innovation in Mountainous Region (Ministry of Education), Institute of Agro-Bioengineering and College of Life Sciences, Guizhou University, Guiyang 550025, China; alihg0519@163.com (A.-L.L.); gzu_zwen@163.com (Z.W.); kyanggz@163.com (K.Y.)

The authors wish to make the following corrections to this paper [1]: In Table A2, of the Appendix, in line 4 of page 14, for the primer of the hpo-miR396b stem-loop RT sequence “GTCGTATCCAGTGCAGGGTCCGAGGTATTCGCACTGGATACGAC**TTCAAG**A” the bold part should be changed to “AAGTTC”.

The authors would like to apologize for any inconvenience caused to the readers by these changes. The changes do not affect the scientific results. 
